# Highlighting Clinical Metagenomics for Enhanced Diagnostic Decision-making: A Step Towards Wider Implementation

**DOI:** 10.1016/j.csbj.2018.02.006

**Published:** 2018-02-27

**Authors:** Jessica D. Forbes, Natalie C. Knox, Christy-Lynn Peterson, Aleisha R. Reimer

**Affiliations:** a Department of Internal Medicine, University of Manitoba, Winnipeg, Manitoba, Canada; b University of Manitoba IBD Clinical and Research Centre, Winnipeg, Manitoba, Canada; c National Microbiology Laboratory, Public Health Agency of Canada, Winnipeg, MB, Canada

**Keywords:** Clinical metagenomics, Diagnosis, Clinical microbiology laboratory, Pathogen detection, Culture-independent diagnostic test, Next-generation sequencing

## Abstract

Clinical metagenomics (CMg) is the discipline that refers to the sequencing of all nucleic acid material present within a clinical specimen with the intent to recover clinically relevant microbial information. From a diagnostic perspective, next-generation sequencing (NGS) offers the ability to rapidly identify putative pathogens and predict their antimicrobial resistance profiles to optimize targeted treatment regimens. Since the introduction of metagenomics nearly a decade ago, numerous reports have described successful applications in an increasing variety of biological specimens, such as respiratory secretions, cerebrospinal fluid, stool, blood and tissue. Considerable advancements in sequencing and computational technologies in recent years have made CMg a promising tool in clinical microbiology laboratories. Moreover, costs per sample and turnaround time from specimen receipt to clinical management continue to decrease, making the prospect of CMg more feasible. Many difficulties, however, are associated with CMg and warrant further improvements such as the informatics infrastructure and analytical pipelines. Thus, the current review focuses on comprehensively assessing applications of CMg for diagnostic and subtyping purposes.

## 1 Introduction

Infectious diseases are a leading cause of morbidity and mortality worldwide. Recent estimates suggest that approximately 19% of global deaths are attributed to infectious diseases [1]. According to the World Health Organization, lower respiratory tract infections are at present, the most common communicable disease causing 3.2 million deaths in 2015; enteric disease and tuberculosis caused 1.4 million deaths each and HIV/AIDS was responsible for 1.1 million deaths [2]. The identification and characterization of pathogenic microorganisms including bacteria, viruses, parasites or fungi that cause infections are critical for the clinical management of patients and the prevention of transmission. In addition, novel, emergent and re-emergent pathogens such as MERS, Ebola, Zika, and the spread of multidrug-resistant pathogens further emphasize the importance of effective diagnostics.

Many syndromes are complicated by the capability of a wide array of pathogens to cause clinically indistinguishable diseases. As a result, accurate diagnosis often requires a battery of traditional microbiological methods such as culture, nucleic acid amplification tests (e.g. polymerase chain reaction; PCR) and serologic assays. Rapid developments have recently been made in the modernization of clinical microbiology laboratories with the employment of multiplex syndromic panels (e.g. BDMax, FilmArray and others), matrix assisted laser desorption ionization-time of flight mass spectrometry (MALDI-TOF MS) and whole genome sequencing (WGS). These methods have played an increasingly important role in clinical microbiology laboratories due to their ability to reduce turnaround time (TAT) from specimen collection to clinically actionable result and through the detection of non-cultivable or fastidious pathogens. However, due to limitations of current diagnostic methodologies (reviewed in [3]) such as requiring a priori knowledge of the pathogen, missed diagnoses occur in 20–60% of cases dependent on the particular syndrome [[4], [5], [6], [7]]. As a consequence, broad-spectrum antibiotics are generally empirically administered, obviating the use of targeted therapies and ultimately resulting in increased mortality along with excess healthcare-associated costs.

**Fig. 1:**
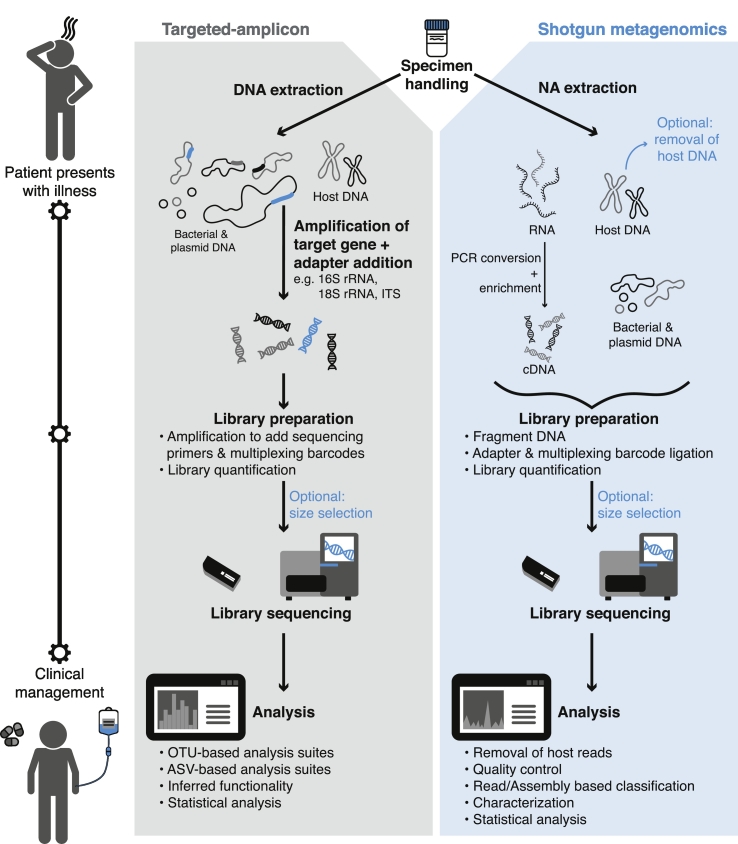
Proposed workflow of a clinical microbiology laboratory using a CMg approach to identify putative pathogens. Patient presents with illness and a biological specimen is obtained. The specimen is subjected to a targeted-amplicon or shotgun metagenomics sequencing workflow. Targeted-amplicon analysis can be accomplished using OTU-based [53,54] or ASV-based [61] software suites. This includes quality filtering, chimera detection, OTU assignment and taxonomic classification via comparison to reference databases [[57], [58], [59]]. Inferred functionality may also be investigated with software such as PICRUSt [143]. Shotgun metagenomics analysis often includes removal of host-associated reads [144,145], sequence data quality control [41,42], read-based classification [48,49] and/or assembly-based classification [[43], [44], [45], [46]], characterization [117,146,147] and statistical analysis. Clinical interpretation of the analysis identifies the putative pathogen followed by conventional confirmatory testing, and the patient is subsequently administered targeted therapeutics. Dependent on the NGS instrument and with continued analytical improvements, time to results can be achieved between 4 and 60 h.

Recent and continuous improvements of next-generation sequencing (NGS) technology have effectively transformed biomedical research. The application of next-generation sequencing (NGS) approaches such as WGS in clinical microbiology laboratories is wide-ranging including for purposes of outbreak management, pathogen surveillance and subtyping and zoonotic transmission determination. Few laboratories are at present applying a culture-independent high-throughput sequencing approach for diagnostic purposes [8]. An NGS-based approach can offer a relatively unbiased pathogen detection through the use of bioinformatics methods and comprehensive reference databases (Fig. 1). From a clinical standpoint, the implementation of clinical metagenomics (CMg) appears to be promising in numerous disciplines including infectious diseases. Thus, CMg has the ability to function as a single assay that can be employed for diagnostic purposes, subtyping, antimicrobial resistance (AMR) detection and virulence profiling. Herein, we discuss the rapidly emerging field of CMg and provide a comprehensive review of NGS culture-independent diagnostic applications thereby describing the potential suitability of this diagnostic assay to be routinely implemented in frontline laboratories.

## 2 What Is Metagenomics?

Metagenomics has previously been used to evaluate the microbial community within a sample or environment, for example, interrogating the gut microbiome and its association with chronic diseases such as inflammatory bowel disease [9], obesity [10] and type 2 diabetes [11]. Analogous to the increased popularity of NGS and bioinformatics, metagenomics is progressively being applied as a novel infectious disease diagnostic assay.

There are two approaches that can be used to examine the microbiome of a given specimen environment, shotgun metagenomics and targeted-amplicon sequencing. Key differences between each approach are described elsewhere [3]. Briefly, shotgun metagenomics attempts to sequence the entire genetic content present in a sample whereas targeted-amplicon represents a more biased approach to a particular group of microorganisms.

**Fig. 2:**
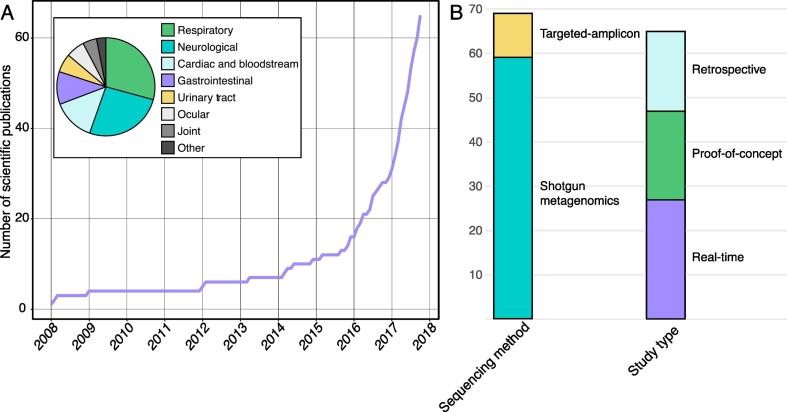
An analysis of reviewed literature. The list of peer-reviewed literature was collated through iterative searches of the National Center for Biotechnology Information's PubMed database and manual curation (see Supplementary Table S1). The following Medical Subject Headings (MeSH; National Institute of Health's (NIH) National Library of Medicine (NLM) controlled vocabulary used to index articles in PubMed) search terms were used in various combinations: metagenom* [MH]; diagnos* [SH]; humans [MH]; clinical metagenomics [tiab]; diagnostics metagenomics [taib]; infectious disease diagnostics [tiab]; not microbiota [MAJR]; not standards [SH]; not review [PT]; not congresses [PT]; not outbreak [tiab]. A) Number of peer-reviewed scientific publications that have used CMg for diagnostic purposes. Pie-graph inset represents the breakdown of reviewed literature according to infection type for CMg diagnosis. B) Bar plot indicates the sequencing method and study type used. An analysis of reviewed literature. The list of peer-reviewed literature was collated through iterative searches of the National Center for Biotechnology Information's PubMed database and manual curation (see Supplementary Table S1). The following Medical Subject Headings (MeSH; National Institute of Health's (NIH) National Library of Medicine (NLM) controlled vocabulary used to index articles in PubMed) search terms were used in various combinations: metagenom* [MH]; diagnos* [SH]; humans [MH]; clinical metagenomics [tiab]; diagnostics metagenomics [taib]; infectious disease diagnostics [tiab]; not microbiota [MAJR]; not standards [SH]; not review [PT]; not congresses [PT]; not outbreak [tiab]. A) Number of peer-reviewed scientific publications that have used CMg for diagnostic purposes. Pie-graph inset represents the breakdown of reviewed literature according to infection type for CMg diagnosis. B) Bar plot indicates the sequencing method and study type used.

Of the two methods, shotgun metagenomics is less taxonomically biased and capable of higher taxonomic resolution as it aims to amplify the whole genomes of every organism present in a specimen. As such, it allows for extended characterization of the microbial population, including subtypes, AMR and pathogenic gene carriage. For this reason, this method tends to lend itself to CMg diagnosis, as can be seen in the proportion of current CMg studies using shotgun metagenomics methodologies (Fig. 2B, Suppl. Table S1). However, there are many issues inherent to shotgun metagenomics; for example, overwhelming quantities of host DNA are often sequenced in comparison to the small fraction of microbial DNA, which is dependent on the biological specimen type. Thus, it can be difficult to obtain high sequence coverage for microbes of interest in specimens where host cells are abundant [12]. In addition, dependent on the sequencing depth required, shotgun metagenomics is significantly more costly than target-amplicon sequencing.

As previously stated, the use of targeted-amplicon sequencing, for pathogen detection is biased due to its inability to query microorganisms across multiple kingdoms (e.g. virus, eukaryotes and prokaryotes) in a biological specimen. In addition, this approach does not provide any additional characterization beyond phylogenetic information. The use of a ubiquitous and taxonomically informative universal genetic marker is used to capture the phylogenetic information of the targeted microorganisms in a given environment. The 16S ribosomal RNA (rRNA) gene, for example, represents the most common marker gene for bacteria and archaea [13]. The gene is ubiquitous in varying copy numbers [14] and provides sufficient sequence variability for taxonomic resolution [15]. Confident taxonomic assignment below the genus level is often difficult due to lack of resolution. Further, accurate classification below the family level of taxonomy has recently been questioned [16]. Several other marker genes with similar limitations to the 16S rRNA gene exist. These marker genes target different taxonomic groups such as the 18S rRNA gene for eukaryotes [17], internal transcribed spacer (ITS) region for fungi [18] and rpoB [19], cpn60 [20], 5S rRNA and 23S rRNA for bacteria and archaea [21,22]. While this method is less suited for an unbiased pathogen detection approach in comparison to shotgun metagenomics, some studies (discussed below) have applied it in a clinical diagnostic setting. Accordingly, we have provided a brief overview of its methods and analytical processes.

### 2.1 Technical Factors Affecting Pathogen Detection in Metagenomics

A metagenomics assay should make every effort to use methodologies that enable nearly complete, unbiased recovery of high quality total nucleic acid content in a sample. Numerous factors can impact the DNA or RNA recovered from a sample including sample handling and storage, nucleic acid extraction, sequencing strategy and even study design [23]. In the context of using CMg for rapid pathogen detection, study design may not be relevant, however, numerous retrospective studies will be necessary to develop validated and robust CMg methods. An extensive review of considerations for metagenomics study design is reviewed elsewhere [24].

### 2.2 Specimen Collection, Handling and Storage

Consistent with basic microbiology methods, aseptic sample collection should be maintained including the use of sterile containers, equipment and solutions used to avoid contamination leading to false positive results. Further, improper sample handling and storage which may be highly specific for particular specimen types has the potential to impact the nucleic acid quality and concentration recovered [23]. For example, studies have described the impact that freeze-thaw cycles have on specimens, including the inability to recover particular groups of bacteria [25,26]. Fouhy et al. [27] also demonstrated that particular storage agents such as glycerol and phosphate buffered solution if used, can impose a bias on the microbial community profile results.

### 2.3 Nucleic Acid Extraction

For shotgun metagenomics and targeted-amplicon approaches, genomic DNA and RNA (shotgun metagenomics only) can be isolated through the use of commercial DNA extraction kits. The amount of genetic material recovered via nucleic acid extraction is highly variable (e.g. varying cell disruption method and inclusion of enrichment steps) [28]. Knudsen et al. [29] and Brooks et al. [30] reported that both the amount of DNA extracted and microbial community profile were affected by the type of commercial DNA kit used and the type of cell disruption method employed. Particular groups of microorganisms such as fungi and parasites are more resistant to cell membrane disruption, thus, modifications to common nucleic acid extraction methodologies may be necessary to ensure sensitive detection of these microorganisms [28]. Dependent on the biological specimen, a large portion of the genomic DNA extracted may be host DNA while the microbial DNA may account for a small fraction. Without a priori knowledge of the expected etiological agent(s), particular laboratory methods may be required to ensure recovery of the pathogen. Viral particles are especially sensitive to this issue due to the diversity of viral genomes (RNA, DNA, circular, linear, double or single stranded) and may require conversion and enrichment of nucleic acid to a form conducive to metagenomics sequencing (Fig. 1). In this context, several studies have highlighted supplementary molecular methods to extract and enrich for viral nucleic acids [23,29,31]. We also direct readers to Greninger et al. [32] for an exhaustive review of the viral metagenomics landscape.

### 2.4 Next-generation Sequencing Instrumentation and Process

Several NGS platforms are currently available with distinct sequencing strategies and features. More detailed information pertaining to current sequencing technologies are reviewed elsewhere [34] Briefly, NGS platforms can be categorized into two major categories: short-read (e.g. Illumina, Ion Torrent) or long-read (e.g. Pacific Biosciences (PacBio), Oxford Nanopore's MinION) sequencing. Illumina short-read sequencers such as the MiSeq and HiSeq are by far, most commonly used for targeted-amplicon and shotgun metagenomics assays, respectively. While the Illumina sequencers range in accuracy between 0.1–1%, run times can be lengthy (e.g. up to 55 h for MiSeq and 84 h for HiSeq) and instrument costs range from moderate to high. The MinION, conversely, represents an attractive alternative and CMg studies are increasingly using the sequencer for diagnostic purposes. The MinION (long-read sequencing) is miniature in size and has the potential for rapid TAT starting from DNA extraction to the acquisition of sequence data in as little as 4 h, though many hurdles need to be addressed prior to widespread implementation including sequencing accuracy, development of robust analytical pipelines for long-read sequencing and higher sequencing cost.

Shotgun metagenomics sequencing processes, particularly for the “gold-standard” Illumina sequencers includes the preparation of a sequencing library. This library consists of extracted nucleic acid prepared for sequencing through a series of vendor-specific enzymatic and mechanical steps. Fragmentation of the nucleic acids into smaller sequences (for short-read sequencing) initially occurs, with an optional size selection step often included to allow a user to eliminate fragment size as a contributing factor in abnormal read length and low sequencing efficiency. The library is finalized for sequencing by the addition of adapters, sequencing primers and multiplexing barcodes and quantification [35]. Many variations and biases can also be introduced at the library preparation stage. For instance, the genomic makeup of microorganisms (GC and repetitive DNA content) can affect genome sequencing coverage and by extension can affect downstream bioinformatics subtyping schemes. These biases are well described in Jones et al. [36].

While similar to shotgun metagenomics, Illumina's protocol for targeted-amplicon sequencing library preparation starts with an initial PCR amplification step to generate amplicons of the targeted genetic marker with adapters. This is followed by the addition of sequencing primers and barcodes and lastly, library quantification and normalization. These steps are described in a schematic workflow in Fig. 1.

### 2.5 Additional Controls and Measures to Mitigate False-positives and -Negatives

Sensitivity and specificity of both targeted-amplicon and shotgun metagenomics sequencing can be enhanced through the inclusion of a few key measures such as inclusion of positive controls (i.e. mock community), no-template control and thorough record keeping. Comprehensive records of each step including date, technician, action/step and lot numbers for reagents used can be critical to help identify sources of variation or contamination in downstream analytics. Negative-controls (also known as no-template controls; DNA-free samples) can be included to ensure all steps starting from sample collection solutions (if used), nucleic acid extraction and sequencing reagents are free of contaminants. If contamination is found, the sequence data from the no-template control will serve as comparator to ensure that the pathogen detected is not a contaminant [37]. It is also imperative that a positive-control such as a mock community DNA or abundant donor sample for which the microbial community profile is well-known is included in the sequencing process. A mock community will contain a mixture of genomic DNA from 10 to 20 microorganisms at varying abundances. Care should be taken to ensure that chosen microorganisms vary in biological factors such as GC content, genome size, Gram stain and ribosomal copy number [38]. This will act as a positive control for wet-laboratory and analytical methods [30] and to test analytical tools to reduce false-positives [38]. Data reproducibility of these positive controls will act as a secondary validation for robustness of methodologies.

Sample type and biomass can also affect sequencing outcomes, such as the increased sequencing bias that occurs in low biomass samples [37]. This may necessitate the need for an enrichment step should the expected abundance of the putative pathogen be near or below the limit of detection (LOD) [39]. Given that no gold standard pipeline exists for metagenomics assays and there are many critical steps whereby bias or variability may be introduced, it is important that both consistency and thorough documentation are followed to ensure that maximal transparency and reproducibility are achieved for reported outcomes [27,40].

Another aspect to consider is the amount of sequencing required to ensure the LOD is achieved for a broad range of infectious agents in the sample type being investigated and that the pathogen signal is discriminatory against ‘background noise’ (e.g. host and other extraneous DNA, normal microbiota DNA and contaminants). These considerations highlight the importance of including a mock community and/or positive-control to ensure the sensitivity and LOD of the method is adequate for the particular specimen, sequencing and analytical strategy being used [28].

### 2.6 Analysis

A bioinformatics analysis of sequence data is necessary to provide a biologically meaningful interpretation of the data, particularly in the case of diagnostics and clinical management. The informatics structure required to run many of the pipelines and software required for metagenomics assays are often extensive and can be computationally expensive, although, this is less of a concern with targeted-amplicon sequence data due to smaller sequence datasets.

#### 2.6.1 Shotgun Metagenomics Analytics

Upon data acquisition, several quality control measures must be undertaken to prepare the data for downstream analytics. The adapter and primer sequences are generally removed as a part of each sequencing platform's software pipeline. Further quality assurance using software such as FastQC [41] can then be used to rapidly identify sequence quality issues including low per base sequence quality, high ambiguous base calls, occasional adapter hold-over and other quality measures. Sequence data quality can be improved through data trimming and the removal of poor quality reads [42]. Should low sequencing coverage ensue, a re-sequencing strategy can then be used. Depending on the NGS instrument and sequencing strategy, other quality control measures may be recommended by the manufacturer and should be followed based on validated workflows.

Two primary approaches to taxonomic profiling of shotgun metagenomics analysis can be employed. These include de novo assembly-based and read-based methods (read mapping and alignment to a reference database). Assembly-based taxonomic profiling generally includes de novo assembly, contig binning and taxonomic/functional profiling. Currently, numerous assembly software are available that can be used to re-construct genomes from metagenomics sequence data including metaSPADES [43] and MEGAHIT [44]. However, re-assembling reads into longer contiguous sequences is challenging due to the overlapping sequence similarity of many microorganisms and it is computationally expensive. Once draft genomes are assembled, software such as CONCOCT [45], MyCC [46] or MetaBat [47] can be employed for contig binning and taxonomic profiling. Conversely, read-based taxonomic profiling generally uses rapid sequence homology searches against reference databases for taxonomic assignment. There are numerous read-based taxonomic profiling software with notable examples including Kraken [48], CLARK [49], Taxonomer [50] and MetaPhlAn2 [51]. While we have listed a few choice software, several others are commonly used. Detailed characteristics of shotgun metagenomics analyses are reviewed elsewhere [24,52]. Each software has strengths and limitations; thus, the choice of which tool to use is largely dependent on the ultimate goal [24].

#### 2.6.2 Targeted-amplicon Analytics

Computational software such as QIIME [53] and mothur [54] are most commonly employed for targeted-amplicon sequence data analysis using operational taxonomic unit (OTU)-based analyses. These software packages incorporate numerous tools such that the majority of the analysis can be executed within a single suite. Similar to shotgun metagenomics, targeted-amplicon analyses include quality control filtering for quality scores, contig (aligned read pairs) length, PCR chimera detection, and presence of homopolymers and ambiguous base calls. Thereafter, contigs are binned into OTUs followed by their taxonomic assignment. In particular, the contigs are clustered into OTUs based on their sequence similarity. In this context, a 97% sequence similarity cut-off is commonly inferred to correspond to the same species [55]. There are several OTU clustering strategies including de novo, closed or open reference. In de novo OTU sequence assignment, contigs are clustered into OTUs based on their sequence similarity to each other. Both closed and open reference align contigs to a reference database followed by OTU clustering based on similarity to the reference sequence, however, contigs that are not matched to a reference sequence are removed in closed reference or clustered de novo in open reference [56]. OTUs are subsequently taxonomically classified utilizing one of several curated reference databases such as greengenes [57], SILVA [58] or RDP [59]. Following classification, community structure as measured via alpha and beta diversity can be examined, in addition to phylogenetic trees, abundance curves or inferred functionality.

A novel approach for the analysis of targeted-amplicon sequence data – amplicon sequence variant (ASV) analyses – has recently been developed [60,61]. These methods have the ability to resolve ASVs to only single-nucleotide differences by sufficiently controlling errors. Attributed to the superior taxonomic resolution, reusability, reproducibility and comprehensiveness, it is expected that ASV analyses will replace OTU-based analyses [60].

### 2.7 Caveats for Data Analysis of Clinical Metagenomics in Pathogen Detection

While the use of CMg as a diagnostic tool is attractive, several analytical caveats and challenges remain prior to widespread implementation. While thoroughly reviewed elsewhere [62] briefly the challenges include: differentiating between colonization and infection, discriminating infectious pathogen from background microbiota, determining live versus dead microorganisms and LODs, quality and breadth of reference databases, and mitigating the mis-interpretation of results as it pertains to false-positive and false-negatives. At present and until these challenges can be overcome, results obtained via CMg assays should be followed by a confirmatory test through molecular, serological, or culture-based methods when feasible.

## 3 Evidence Supporting Clinical Metagenomics in Diagnostic Laboratories

The employment of a metagenomics approach in a clinical setting (i.e. CMg) has the potential to rapidly identify and characterize putative pathogens. Presentation and symptoms of infections are often not specific to a particular pathogen and may result in a broad differential diagnosis [63]. Diagnosis is further complicated by the difficulty in discerning viral and bacterial infections. Often, clinicians empirically treat the patient with antivirals, antibacterials or both while awaiting microbiologic results [63]. Multiplex PCR panels for the detection of bacterial, viral and some other pathogens are now routinely used for diagnostic purposes due to their sensitivity, speed and cost effectiveness [64]. FDA-approved pathogen panels include Biofire FilmArray, Luminex NxTAG, Luminex Verigene and GenMark eSensor. However, many pitfalls are associated with these assays such as the potential for cross-reactivity between microorganisms with high sequence homology or inability to detect rare isolates. Hence, there is a growing need to implement diagnostic assays that overcome these and other limitations (reviewed in [64]) to detect the causative pathogen with high sensitivity and ultimately provide effective clinical management and infection control measures. In this regard, CMg has the potential to revolutionize diagnostics. Further exploration however must be conducted to investigate the ability of CMg to detect emergent pathogens or novel genetic variants such as that observed with the SARS epidemic [65].

Reduced costs, TAT and increased sensitivity will promote the incorporation of CMg in clinical practice in the near future for various syndromes. Thus, CMg has the potential to revolutionize diagnostic microbiology. Though the field of CMg is in its infancy, several successful applications of this technology have been reported. To date (November 2017), approximately 65 studies have applied CMg for pathogen detection purposes (Fig. 2; Suppl. Table S1). As summarized in Fig. 2A, the numbers of CMg reports are exponentially increasing. Of available literature, CMg assays have been most commonly used for diagnostic purposes in respiratory (29.2%), neurological (26.2%), cardiac and bloodstream (13.9%) and gastrointestinal (10.8%) infections (Fig. 2). Fig. 2B shows that most CMg studies have employed shotgun metagenomics. In addition, there are a fairly equal number of studies that have used CMg for real-time diagnosis, retrospective diagnosis and as a proof of concept. The following sections describe reports where CMg has been employed.

### 3.1 Respiratory Illness

As indicated previously, respiratory illnesses are a principal cause of morbidity and mortality globally [2]. Viral infections in particular are pervasive in a wide array of hosts, affect persons of all ages and account for many healthcare associated infections [66,67]. One of the first identifications of respiratory pathogens via a CMg approach was in 2009 [68]. This study included both nasopharyngeal aspirates and fecal specimens collected during a large-scale norovirus outbreak and concurrent seasonal influenza A. Applying metagenomics, influenza reads were detected in each (n = 3) nasopharyngeal aspirate and were sufficient for subtyping. Concomitantly, norovirus was detected in 4 of 5 fecal specimens with 78–98% coverage. While few studies adapting similar methodologies were performed in the following years [[69], [70], [71]], publications in the use of metagenomics for the detection of pathogens from respiratory specimens increased considerably in 2016 [[72], [73], [74], [75], [76]]. CMg in respiratory illnesses has now been used: as a means of retrospective diagnosis; applied when standard microbiological testing has proven inconclusive; used in real-time; and used for outbreak detection.

Pendleton et al. [77] reported two cases where real-time CMg was used to diagnose cases of pneumonia. In the first case, BAL fluid was concurrently sent for standard microbial testing, shotgun metagenomics and 16S rRNA targeted-amplicon sequencing on the MinION platform. Nine hours post sampling, shotgun metagenomics results revealed a sequence that aligned to *P. aeruginosa*. Culture-based confirmatory testing was available 14 h later. The 16S rRNA targeted-amplicon community profiling revealed a low-diversity community that was dominated by *P. aeruginosa* (65% relative abundance). In a second case, the MinION platform was used to sequence BAL fluid; 6 sequences between 909 and 8288 bp aligned to *Staphylococcus aureus*. Microbiological testing later reported >10^4^ CFU of *S. aureus*. Targeted-amplicon profiling revealed a community dominated by *Staphylococcus* sp. at a 95% relative abundance. These studies exemplify the promise of providing clinically actionable results via CMg.

Though respiratory illness can be caused by a variety of microorganisms, viruses are the most common culprits. Yan et al. [78] reported a case of a 60-year-old female who presented to the hospital with severe community-acquired pneumonia (CAP). Extensive microbiological testing was negative. Subsequently, BAL fluid and throat swab specimens were subjected to shotgun metagenomics sequencing using the Ion Torrent platform. Sequencing of the BAL fluid revealed, human rhinovirus B91 as the most abundant microorganism, accounting for 58.8% of reads while *Acinetobacter baumannii* and *S. pneumoniae* were present at lower levels (17.1% and 0.7%, respectively). BAL metagenomics results were similar to those reported from the throat swab. Confirmatory culture-testing suggested that human rhinovirus B91 was the etiological agent. The patient improved and was discharged 18 days post symptom onset. Gong et al. [79] demonstrated the ability to detect viruses that were missed by routine microbiological testing. The study included 40 respiratory specimens such as throat swabs, nasopharyngeal swabs, or sputum from children diagnosed with unknown causes of acute bronchiolitis, croup, or respiratory tract infections. Seven respiratory viruses were detected in 26 of 40 specimens. Other studies have also explored the applicability of CMg to diagnose viral respiratory infections where microbiological testing resulted in missed diagnoses [73,76,[80], [81], [82]]. One study in particular reported a Rhinovirus diagnosis from a PCR assay in a transplant recipient early in illness [71]; shotgun metagenomics correctly identified the causative pathogen as human Enterovirus C104. Reasons for the initial incorrect diagnosis are attributed to the cross-reactivity issues of the multiplex PCR assay utilized.

Comparatively, recent studies have applied CMg to survey respiratory specimens for less commonly associated pathogens such as bacteria [12,74] and fungi. For example, ventilator-associated pneumonia (VAP) represents one of the most common nosocomial infections in the intensive care unit and affects one third of patients admitted for non-infectious ailments that require mechanical ventilation [83]. Pathogen detection via CMg in three cases of VAP was recently reported [74]. In each case, shotgun metagenomics results were concordant with culture-based tests and were able to detect additional putative pathogens. Further, AMR genes identified through basic local alignment search tool (BLAST) analysis were consistent with antimicrobial susceptibility testing (AST). Of note, culture results from one patient were inconclusive, hence providing evidence for increased sensitivity of CMg over culture.

Numerous studies have described pathogen detection from respiratory specimens through a CMg approach as superior to standard microbiological testing due to the lower TAT, limit of detection (LOD) and unbiased sequence targets. Hilton et al. [75] analyzed the efficacy of a number of diagnostic approaches for VAP. Two high-throughput approaches – 16S rRNA targeted-amplicon and shotgun metagenomics – were evaluated and compared to standard microbiological testing including culture. Results were concordant in 5 of 6 specimens between shotgun metagenomics and culture whereas results of only 2 specimens were consistent between the targeted-amplicon assay and culture. A more recent study compared the detection of respiratory virus-positive and undiagnosed nasopharyngeal swabs from pediatric patients between a shotgun metagenomics approach and an FDA-cleared respiratory virus panel (GenMark eSensor) [72]. Metagenomics was able to accurately detect the known viral pathogen in 86% of specimens and from undiagnosed specimens, metagenomics agreed with the respiratory virus panel in 93% of specimens. Additionally, 12 viruses were detected via sequencing that were not detected with the panel. This result is either due to the panel not targeting those viruses or divergent/variant genome sequences. Lastly, the authors reported that partial or full-length viral genomes were recovered in 86% of respiratory virus-positive samples thus extending analysis to determine antiviral resistance, subtyping and phylogenetic assessments. Similarly, Yang et al. [69] reported agreement in results, particularly the detection of known respiratory viral pathogens, between shotgun metagenomics and PCR in 15 of 16 specimens. Notably, CMg outperformed PCR testing in one specimen whereby a coinfection was observed.

#### 3.1.1 Methodological Studies

While several metagenomics protocols have been optimized, validated or published in recent years [8,84] one study reported the use of Oxford Nanopore's MinION sequencing device for rapid detection of bacterial pathogens in a variety of biological specimens [85]. The authors tested their protocol against a mock community and a pleural effusion from a patient with empyema. Shotgun metagenomics results were compared between IonPGM and the MinION; 16S rRNA targeted-amplicon sequencing with IonPGM was also performed. *Prevotella* spp. which have previously been implicated in infections of the respiratory tract [86] were identified as the major taxon according to each sequencing methodology but undetected via culture. BLAST [87,88] analysis against the GenomeSync database [89] revealed *P. oris* as the dominant species. Likewise, *Streptococcus anginosus*, was similarly reported via each approach and verified via culture. A second study also compared the application of CMg between two sequencing platforms, Roche 454 (discontinued) and Illumina's MiSeq on a nasopharyngeal swab that was positive to the 2009 pandemic influenza A H1N1 strain [90]. Though differences were apparent in terms of depth and coverage, both shotgun metagenomics and targeted-amplicon approaches were able to detect the H1N1 virus as well as *Streptococcus pneumoniae*.

While the interest in CMg is largely based on the ability to rapidly detect pathogens from clinical specimens, Langelier et al. [91] has also shown that it is possible to detect both microbial and human RNA to ultimately allow for concurrent transcriptional profiling of the host immune response hence permitting precision medicine and infectious disease diagnostics. This study included a cohort of 22 hematopoietic cellular transplant recipients with acute respiratory illness who underwent both bronchoscopy and BAL. Standard microbiological testing was performed including quantitative cultures, PCR and other diagnostic assays. BAL was subjected to DNA and complementary DNA (cDNA) sequencing via paired-end Illumina sequencing. Microbiological testing detected microorganisms in 7 of 22 patients, 6 of which were considered pathogens. Further, shotgun metagenomics also detected known respiratory pathogens in 6 patients with negative microbiological testing including human coronavirus 229E and human rhinovirus A. Interestingly, while common causes of CAP these viruses are not included on many respiratory viral PCR panels. *Streptococcus mitis* and *Corynebacterium propinquum*, both virulent pathogens were detectable in one patient. Several DNA viruses were also identified and analyses of cDNA shotgun metagenomics sequencing provided evidence of active replication for some. The host airway immune response was also evaluated; significantly increased expression of immune response genes was detected in patients with confirmed lower respiratory tract pathogens. Thus, this study demonstrates that CMg has substantial ability to accurately detect pathogens and these results can be utilized to inform the host immune response.

#### 3.1.2 Potential Use in Respiratory Outbreaks

The first investigation of this assay's use in a respiratory outbreak of human parainfluenza 3 virus was only recently described [33]. Specimens from 3 patients with hospital-acquired human parainfluenza 3 and 10 temporally associated community-acquired human parainfluenza 3 were included in the analysis. TAT from specimen collection to actionable result was <60 h. Each specimen included in the analysis had a minimum of 1 read corresponding to the human parainfluenza 3 genome and full genome coverage was achieved in 8 of 13 specimens. Phylogenetic clustering revealed identical genome sequences in two of the hospital-acquired cases whereas there was insufficient sequence coverage for the third hospital-acquired specimen. Hence, this study provides evidence that CMg has the potential to extend its utility beyond the clinic to public health activities such as outbreak detection and investigation.

### 3.2 Infections of the Central Nervous System

Though central nervous system (CNS) infections have serious implications for the recovery of a patient due to the sheer volume of testing that is often required, >50% of cases are undiagnosed [7]. Therefore, it is understandable that numerous successful applications of CMg to diagnose neurological infections have been reported in recent years [[92], [93], [94]]. Notably, from a diagnostic perspective, this methodology was first applied to a case of a 14-year-old male with severe combined immunodeficiency who presented to a medical facility 3 times with complaints of fever and headache that subsequently advanced [92]. Repeated routine diagnostic workup was inconclusive, though an MRI eventually identified an encephalitis-like condition. Shotgun metagenomics using the Illumina platform was ultimately performed on cerebrospinal fluid (CSF) and serum specimens. Within 48 h of specimen receipt, results of CSF detected 475 of 3,063,784 sequence reads (0.016%) matched most closely to the *Leptospira borgpetersenii* genome. Targeted antimicrobial treatment with intravenous penicillin G was administered. PCR and serologic testing eventually confirmed a neuroleptospirosis diagnosis. As a result of the targeted treatment, the patient's condition improved and was discharged 32 days later. Several additional case studies have been published using a similar CMg pipeline to successfully diagnose rare, emergent or re-emergent etiologies of encephalitis. These include the diagnosis of neuroinvasive astrovirus infection in an immunocompromised patient with encephalitis [95], meningoencephalitis caused by hepatitis E virus in a lung transplant recipient [96], *Balamuthia mandrillaris* encephalitis [97], St. Louis encephalitis virus [98], neurobrucellosis [94], Cache Valley virus [99] and West Nile virus [93]. In one study in particular, a long TAT due to staffing levels resulted in a delay of nearly a month to receive a diagnosis via CMg. A shorter TAT would have resulted in a much different treatment plan for the patient, specifically obviating the need for a wide array of antimicrobials and the potential to enhance immunosuppression [93].

Further studies using various metagenomics analytical pipelines have also successfully diagnosed neurological infections. In many cases, the researchers were able to use CMg not only to identify the causative agent but also to gain insights into disease progression. Wylie et al. [100] applied shotgun metagenomics to detect a rare infection of *Propionibacterium acnes* in an immunosuppressed patient with chronic meningitis. Subsequent sequencing of specimens during and following treatment demonstrated a decrease of *P. acnes* to background levels. The CSF of a female patient in Denmark with severe encephalitis was subjected to shotgun metagenomics after standard microbiological testing failed to detect any causative agent [101]; human pegivirus, a viral agent previously attributed to asymptomatic infections, was the only pathogen detected in serum and CSF. CMg has also been applied to confirm PCR detection and further, to rule out immune escape mutants as the causative agent of relapse in a case of Ebola virus causing meningoencephalitis [102]. A further study which identified a rare opportunistic pathogen, *Psychrobacter* sp., by shotgun metagenomics was also able to identify several virulence factors [103]. Mai et al. [104] successfully detected Japanese encephalitis virus in a urine specimen via shotgun metagenomics and proposed urine as a valuable diagnostic specimen in flavivirus-endemic regions. Combined, these studies suggest that CMg has the potential to be an extremely valuable tool in the case of rare diseases.

Pathogen detection by CMg is able to positively influence clinical decisions and in some cases with TATs much shorter than standard microbiological testing. In 2015, Perlejewski et al. [105] identified human herpes virus 1 in the CSF of a patient that was negative for serology; seroconversion occurred with subsequent sampling. Further, Powassan virus was detected in a patient with severe and progressive encephalitis via shotgun metagenomics nearly 30 days prior to serologic results [106]. A prospective study was undertaken at Johns Hopkins University to determine the feasibility of shotgun metagenomics in CNS infection diagnosis [107]; of the 10 patients enrolled in the study, CMg contributed to the clinical outcomes of 8 patients. Both bacterial and viral infections were detected as the causative agent, along with cases where infection was ruled out. In one patient, shotgun metagenomics was able to identify *Mycobacterium tuberculosis* when traditional testing failed to identify any agent; rapid response to anti-tuberculosis treatment was observed. Although CMg was unable to identify with precision the infectious agents in the remaining cases, it contributed to the understanding of neuropathologic processes in 5 other cases.

### 3.3 Cardiac and Bloodstream Infections

There is a need to accelerate the diagnosis of cardiac and bloodstream infections (BSIs). Particularly in septic cases, appropriate clinical decision-making should be provided within 6 h in order to reduce morbidity and mortality [108]. Though culture can be lengthy and laborious, it remains the gold standard. Furthermore, approximately 50% of BSI cultures are reported as culture-negative thus delaying appropriate clinical management [109]. Detection may be impeded via early and empirical use of antibiotics (prior to obtaining biological specimens), due to the presence of non-culturable or fastidious microorganisms or rather, low circulating numbers of the causative agent. Molecular diagnostics have proven to be an important complement to culture; nonetheless, molecular diagnostic improvements are still needed.

CMg has proven useful in cases of infective endocarditis [110,111] and idiopathic pericarditis [112]. Imai et al. [110] performed a shotgun metagenomics assay in 3 cases of infective endocarditis, two of which were culture-positive for *Enterococcus faecalis* and *Streptococcus mutans*. Shotgun metagenomics was conducted using the Illumina MiSeq and taxonomies were assigned using the National Center for Biotechnology Information's (NCBI) taxonomy database. While CMg results were concordant for the two culture-positive specimens, shotgun metagenomics detected, *S. sanguinis* in the culture-negative specimen. The identification of Gram-positive cocci from the patient's valve tissue via Gram-stain confirmed the result.

Accurate BSI diagnosis via culture is further complicated in cases of polymicrobial infections. In this context, CMg assays for the identification of polymicrobial bacterial DNA in blood have been explored [113]. Lelouvier et al. [114] retrospectively examined the blood microbiota profiles of 100 patients admitted to the hospital for acute coronary syndrome and 100 controls at high cardiovascular risk free of coronary disease using a 16S rRNA targeted-amplicon approach. Interestingly, results highlighted one patient as having an unusually high quantity of bacterial DNA; these reads mapped to two genera which have been reported in extreme BSI cases and to another genus which has not yet been reported to be associated with BSIs. Another retrospective study included 75 children (median age of 15 months) from Burkina Faso with severe febrile illness [115]. Standard blood culture and malaria testing were conducted at time of admission and 16S rDNA targeted-amplicon sequencing and analysis was performed. Results revealed a higher sensitivity, outperforming culture by diagnosing 22 patients compared to 12 via culture. Interestingly, this study also provided evidence that patients with concurrent acute malaria or those in recovery had a 7-fold increased risk of presenting with a polymicrobial BSI. An additional study explored the use of shotgun metagenomics on free circulating plasma DNA from 7 septic patients [116]. In each case, CMg and blood culture results were concordant and identification of pathogenic species from culture-negative samples was also possible. In addition, reads were aligned to the CARD database [117] thereby allowing for the identification of AMR genes. These results were confirmed by standard microbiological testing. Successful virus detection via CMg in serum has also been described, particularly with Zika virus [118], human herpesvirus 6 [119] yellow fever virus [120] and Ebola [121]. In these studies, CMg and routine microbiological testing results were either concordant or CMg outperformed.

### 3.4 Enteric Disease

Diagnosis of enteric infections, historically have been dependent on culture. In recent years however, there has been a dramatic uptake of culture-independent diagnostics such as multiplex PCR panels in clinical microbiology laboratories [122]. Due to a low pathogen discovery rate (60% [5]) it is unsurprising that CMg has been applied as a diagnostic aid for enteric pathogens for nearly a decade. In 2008, Nakamura et al. [123] conducted one of the first proof-of-concept studies to employ shotgun metagenomics to detect bacterial pathogens. In this case, a 34-year-old male presented with severe gastrointestinal symptoms following the consumption of undercooked chicken. While culture for possible enteric pathogens and real-time PCR (RT-PCR) for norovirus did not reveal the pathogen, metagenomics identified 156 *Campylobacter jejuni* sequences followed by confirmatory testing using *Campylobacter*–specific PCR.

More recently, CMg has been employed to detect the first case of gastrointestinal basidiobolomycosis [124]. A 41-year-old HIV-positive female from Cameroon presented to the hospital with a 2-month history of abdominal pain. A sizable (inflammatory) mass was seen on her abdomen by CT-scan and histopathological examination revealed large hyphae that were encased by a thick eosinophilic material. Sanger sequencing of the ITS results were inconclusive however, ultra-deep pyrosequencing of the ITS2 region revealed 4 fungal species with *B. meristoporus* representing 80% of the sequences. The authors concluded that this approach is ready to be integrated into clinical microbiology laboratories to improve infectious disease diagnostics and patient care.

Much research has been aimed at optimizing and validating CMg assays. Several studies in recent years have assessed the diagnostic potential of CMg in enteric disease and how results might be different from conventional methodologies. Zhou et al. [125] examined the concordance between various diagnostic tools such as PCR, qPCR, 16S rRNA targeted-amplicon and shotgun metagenomics assays specifically for *Clostridium difficile* infection. Targeted-amplicon assays outperformed shotgun metagenomics identifying *C. difficile* in PCR and qPCR positive samples (90.9% vs. 86.3%, respectively), likely attributed to read depths. Several other pathogens (e.g. norovirus and sapovirus) were similarly detected via shotgun metagenomics. However, this study also suggests that sensitivity improvements are required. A second study examined the efficacy and concordance between shotgun metagenomics, microscopy and multiplex PCR on 4 stool specimens obtained from patients with persistent diarrhea [126]. Metagenomics detected 8–11 potential enteric pathogens in all samples. High diagnostic agreement of bacterial pathogens was reported between PCR and shotgun metagenomics; however, shotgun metagenomics identified some pathogens not detected by PCR. Moreover, microscopy outperformed shotgun metagenomics in the detection of helminth and protozoan pathogens, hence reflecting current sensitivity issues with CMg assays to particular microorganisms. Andersen et al. [127] assessed the sensitivity of a shotgun metagenomics assay, testing 22 *Campylobacter* culture-positive and 5 culture-negative specimens spiked with *C. jejuni*. Higher spiking levels were associated with more hits, though no linear correlations were observed. The sensitivity of shotgun metagenomics has clearly improved and is now clinically relevant though as discussed above, challenges still remain.

Though most CMg studies have employed Illumina sequencing, a recent study investigated the use of the Oxford Nanopore's MinION sequencing device and the NanoOK RT [128] software package as a tool to detect pathogens [129]. One aim of the study was to detect bacterial enteric pathogens from fecal specimens of preterm infants at risk of sepsis and necrotizing enterocolitis. Pathogenic taxa such as *K. pneumoniae* along with AMR profiles could be identified. The authors also concluded that the time from specimen collection to tailored treatment could be as little as a few hours.

### 3.5 Ocular Infections

Few studies have investigated the application of CMg to diagnose ocular infections [[130], [131], [132], [133]]. The first proof-of-concept investigation of ocular infections included 5 subjects with known etiologies and 1 subject with an unknown cause of bilateral chronic uveitis [130]. Shotgun metagenomics results were concordant with standard microbiological testing results in 4 of 5 known etiology cases; rubella virus was detected in the idiopathic case, which was in agreement with the patients' medical history A second study from the same group included specimens that were PCR-negative (n = 36) or PCR-positive (n = 31) for several pathogens (herpes simplex virus 1 and 2, cytomegalovirus, varicella-zoster virus and *Toxoplasma gondii*) and evaluated the use of shotgun metagenomics for pathogen detection [131]. Pathogens were identified in 87% of PCR-positive specimens and 6 different pathogens in 22% of the PCR-negative specimens. Recently, Gao et al. [132] employed shotgun metagenomics in a case where all routine diagnostic approaches failed, ultimately diagnosing a rare case of Malayan filariasis from the Unites States. Diagnosis was subsequently validated by the observed therapeutic efficacy. These studies demonstrate the benefits of detecting pathogens missed in routine diagnostics.

### 3.6 Urinary Infections

To date little research has described the use of CMg to diagnose urinary infections. Siddiqui et al. [134] reported a case of a 61-year-old female with overactive bladder syndrome (not associated with microorganisms) and a 10-year history of urinary tract symptoms. Culture revealed significant bacteriuria caused by viridans group streptococci. Consistent with culture, 16S rRNA targeted-amplicon sequences assigned to α-hemolytic *Streptococcus* were identified. Moreover, numerous fastidious microorganisms were also detected, such as *Atopobium* spp. and *Ureaplasma* spp., thus indicative of a polymicrobial state. Though a course of antibiotics was applied, the patient returned a year later with similar presentation; the culture was negative though the microbial profile via sequencing was similar to the initial profile.

A recent study collected urine samples from all patients suspected of having a urinary tract infection (UTI) over a 2-day period (n = 41) [135]. All culture-positive samples contained >200 ng DNA. AMR determination via shotgun metagenomics correctly predicted the resistance phenotype, which was confirmed by routine AST and isolate WGS in 20 of 32 cases. Schmidt et al. [136] investigated the utility of MinION sequencing to detect bacterial pathogens in urine specimens. Specifically, 10 urine specimens with bacterial DNA enrichment and 5 healthy urines that were spiked with *E. coli* were sequenced. MinION sequencing was able to correctly identify pathogens and identify AMR genes. The MinION sequencing results were also compared to Illumina sequencing; Illumina reported 55 AMR genes whereas the MinION reported 51. The authors concluded that the time needed for the MinION assay is comparable to PCR (approximately 4 h).

### 3.7 Joint Infections

Ruppé et al. [137] conducted a proof of concept study that used shotgun metagenomics on 24 bone and joint infection (BJI) specimens to identify pathogens and determine AMR profiles. Specimens were defined as monomicrobial (n = 8) or polymicrobial (n = 16) via culture. Culture and shotgun metagenomics results were concordant in each monomicrobial case for identifying the pathogen. Using metagenomics for polymicrobial cases, however, resulted in only 58.2% of bacteria being classified at species level while this sensitivity increased to 74.5% at the genus level. Difficulties associated with the identification of polymicrobial infections are in part based on incomplete or variably curated databases. A total of 273 bacteria that were not detected in culture were reported by shotgun metagenomics, of which 182 were possible pathogens. In the context of antibiotic susceptibility, accurate predictions were inferred in 94.1% of monomicrobial and 76.5% of polymicrobial cases.

Diagnosis of prosthetic joint infections has also been explored via a CMg approach [138]. A 52-year-old male with a chronic right knee prosthetic joint infection was referred for evaluation to the Department of Orthopedic Surgery at Mayo Clinic. The patient had an extensive medical history and all microbiological testing including bacterial, fungal and mycobacterial culture and PCR, were negative. Sonicate fluid from the patient's initial joint reconstruction was subjected to shotgun metagenomics. Using Kraken [48], results suggested *Mycoplasma salivarium* as the plausible pathogen. A 16S rRNA targeted-amplicon assay was performed on the same specimen. The strongest match showed 99.8% similarity to *M. salivarium* strain PG20. Validation tests including selective culture of sonicate fluid were unsuccessful. Nine months following reimplantation, the patient returned reporting symptom reoccurrence. Resampling and a 16S rRNA target-amplicon assay again indicated *M. salivarium* and the patient was treated with doxycycline. This case is extremely important as it describes the identification of difficult to detect pathogens using standard microbiological testing. A subsequent study assessed the application of shotgun metagenomics of 97 sonication fluid specimens and evaluated these results compared to routine aerobic and anaerobic culture [139]. Fifty derivation samples were used to determine the LOD. Specifically, optimal thresholds were determined based on the number of bacterial reads that corresponded to infection. These thresholds were confirmed in 47 validation samples. Compared to culture, species-level metagenomics sensitivity was 88% (derivation – 92%, validation – 84%) and genus-level sensitivity was 93%. Hence, these results confirm the possibility of rapid accurate diagnostics for joint infections using CMg.

### 3.8 Other Cases of Interest

In a noteworthy case, 3 patients who received visceral-organ transplants from a single donor died of febrile illness within 6 weeks of transplantation [140]. Routine microbiological testing results were inconclusive and included culture, PCR, serologic assays and microarray. RNA from both the liver and kidney recipients were subjected to shotgun metagenomics where sequences corresponding to a new arenavirus were detected. Analysis revealed that this virus was closely related to lymphocytic choriomeningitis virus. Results were validated via sequence-specific PCR and confirmed the presence of the virus in the kidneys, liver, blood and CSF of the transplant recipients. In addition, immunohistochemical analysis identified antigens in the liver and kidney. Lastly, immunoglobulin M and immunoglobulin G antiviral antibodies were detected in the donor's serum.

The application of CMg to identify bacterial infections and determine AMR in cases of acute cholecystitis has also been explored [141]. Bile, stool and salivary specimens from 6 patients who underwent cholecystectomy were subjected to shotgun metagenomics; single or multiple bacterial infections were identified in 4 cases and additionally, extended-spectrum β-lactamase genes were identified in 2 bile specimens. These results were validated via WGS.

## 4 Summary and Outlook

The prospect of broad-range pathogen detection by CMg has been demonstrated in various studies and clinical contexts. Despite its utility, several universal challenges need to be addressed prior to widespread implementation. Technical challenges include, but are not limited to: specimen complexity, generating appropriate pipelines, and high-quality and discriminatory databases, determining appropriate LODs for different pathogens and specimens, and interpreting the presence of opportunistic pathogens. These challenges are nonetheless not a major barrier, as they will be overcome through continued research and development, including NGS technology improvements, in addition to retrospective and prospective applications. Prior to routine use of CMg in the clinical microbiology laboratory, further considerations must also be addressed including: the cost to implement, cost per test, personnel training, standardization of CMg methodologies and data analysis, diagnostic accreditation, and methods to determine clinical relevance along with interpretation guidelines for clinicians.

Reduced costs and instrument footprint combined with improved methodologies have positioned CMg within the scope of clinical microbiology laboratories. Though wet-laboratory workflows and data analysis pipelines combined with regulatory requirements are rapidly evolving, continued improvements are nonetheless needed. International conferences have recently been developed to discuss key aspects related to CMg [142]. Thus, as we move towards a CMg era, diagnostic laboratories should consider implementing the assay into practice particularly when standard microbiological testing fails to identify the putative pathogen. We anticipate that within the next 10 years, CMg will be a widely used tool in the clinical microbiology laboratory.

## Abbreviations

AMR: antimicrobial resistance
AST: antimicrobial susceptibility testing
ASV: amplicon sequence variant
BAL: bronchoalveolar lavage
bp: base pair
BJI: bone and joint infection
BLAST: basic local alignment search tool
BSI: bloodstream infection
CAP: community-acquired pneumonia
cDNA: complementary DNA
CFU: colony forming units
CMg: clinical metagenomics
CNS: central nervous system
CSF: cerebrospinal fluid
DNA: deoxyribonucleic acid
GC: guanine-cytosine
HIV: human immunodeficiency virus
ITS: internal transcribed spacer
LOD: limit of detection
MALDI-TOF MS: matrix assisted laser desorption ionization-time of flight mass spectrometry
MRI: magnetic resonance imaging
NCBI: National Center for Biotechnology Information
NGS: next-generation sequencing
OTU: operational taxonomic unit
PacBio: Pacific Biosciences
PCR: polymerase chain reaction
qPCR: quantitative polymerase chain reaction
RNA: ribonucleic acid
RT-PCR: real-time polymerase chain reaction
rRNA: ribosomal ribonucleic acid
TAT: turn-around-time
UTI: urinary tract infection
VAP: ventilator-associated pneumonia
WGS: whole genome sequencing

The following is the supplementary data related to this article.

Table S1 Summary of current clinical metagenomics studies. Table S1

## Conflict of Interest

The authors declare no conflicts of interest.
