# Supplementary material for: Highlighting Clinical Metagenomics for Enhanced Diagnostic Decision-making: A Step Towards Wider Implementation
Source: Comput Struct Biotechnol J. 2018 Feb 27;16:108–20. doi: 10.1016/j.csbj.2018.02.006 (PMC6050174; doi:10.1016/j.csbj.2018.02.006)
Supplement: Table S1 — Summary of current clinical metagenomics studies. [file mmc1.docx]

| **Table S1.** Summary of current clinical metagenomics studies. | | | | | |
| --- | --- | --- | --- | --- | --- |
| **Infection type** | **Sequencing method** | **Confirmatory testing** | **Outcome** | **Year of Publication** | **Reference** |
| **Real-time** | | | | | |
| Cardiac and bloodstream | Shotgun | No specific confirmatory testing is mentioned, though the viral genome was closed via PCR | The identification of yellow fever virus in one patient led to the etiological agent and directed response to a disease outbreak in Uganda. | 2012 | [120] |
| Cardiac and bloodstream | Shotgun | Culture | Identification of causative agent in infective endocarditis in 3 patients (one of which was culture negative) | 2014 | [110] |
| Cardiac and bloodstream | Shotgun | N/A | Identification of fastidious *Abiotrophia defectiva* in infective endocarditis; patient underwent resection surgery and a long term course of antibiotics, the patient recovered with no relapse at time of publication | 2015 | [111] |
| Gastrointestinal | Shotgun; Targeted 16S rRNA | Culture; 16S rRNA PCR; WGS; AST; | Identification and AMR profiling of *Klebsiella pneumoniae* as causative agent in necrotising enterocolitis | 2017 | [129] |
| Gastrointestinal | Targeted ITS | Histopathology | Identification of *Basidiobolus meristosporus*, a very rare and emergent fungal infection, as the causative agent in a patient presenting with abdominal pain; following surgery the patient received targeted therapy but subsequently died after several episodes of septic shock | 2017 | [124] |
| Joint | Shotgun; Targeted 16S rRNA | 16S rRNA PCR | Identification of *Mycoplasma salivarium* as the causative agent in prosthetic joint infection; patient underwent re-implantation surgery but reported relapse at 9 months post-surgery, patient was being treated for *M. salivarium* infection at time of publication | 2017 | [138] |
| Neurological | Shotgun | Targeted PCR | Identification of *Leptospira santarosai* as causative agent in febrile illness; upon receiving metagenomics results (and prior to confirmatory test results) patient was started on targeted treatment for leptospirosis, patient recovered over several weeks and returned home close to premorbid state | 2014 | [92] |
| Neurological | Shotgun | Pathology; targeted PCR | Parallel metagenomics testing of samples around patient time of death confirmed presence of *Balamuthia mandrillaras* in patient with meningoencephalitis | 2015 | [97] |
| Neurological | Shotgun | *In situ* hybridization of brain tissue using probes derived from the Human astrovirus capsid | Identification of human astrovirus in a case of progressive encephalitis; despite a lack of approved therapies, the patient was treated with antivirals though did not response and eventually passed away 4 months after the metagenomics diagnosis | 2015 | [95] |
| Neurological | Shotgun | 18S rRNA PCR; immunohistochemistry | Identification of *Balamuthia mandrillaris* in a case of endophthalmitis progressing to meningoencephalitis; patient was diagnosed around the time patient was put on comfort care and later expired | 2015 | [148] |
| Neurological | Shotgun | Lawrence-Livermore pan-microbial assay; targeted PCR | Metagenomics was used to get typing information of human pegivirus infection in a case of severe encephalitis | 2016 | [101] |
| Neurological | Shotgun | RT-PCR; Serology | Metagenomics was used to sequence the ebola virus genome of a patient with late relapse causing meningoencephalitis to check for immune escape mutants | 2016 | [102] |
| Neurological | Shotgun | Targeted PCR; Serology | Identification of *Brucella* spp. by metagenomics in a patient originally diagnosed with antibiotic resistant tuberculosis; the patient was put on targeted antimicrobial therapy for neurobrucellosis and reported her symptoms had resolved two weeks later | 2016 | [94] |
| Neurological | Shotgun | Repeated metagenomics sampling of patient during diagnosis and treatment, and sampling of control patients | Identification of *Propionibacterium acnes* in chronic meningitis of an allogeneic stem cell transplant patient; patient received targeted treatment and repeated metagenomics sampling showed a decrease in *P. acnes* reads to background level | 2016 | [100] |
| Neurological | Shotgun | Repeated metagenomics sampling of patient; RT-PCR; viral culture | Metagenomics identified St. Louis encephalitis virus in a patient; due the patient’s diagnosis and poor prognosis, the patient was put on comfort care and died the next day | 2017 | [98] |
| Neurological | Shotgun | RT-PCR; Serology | Identification of Japanese encephalitis virus in a urine sample of a patient with sudden encephalitis; this was the first reported case of JEV detection in urine | 2017 | [104] |
| Neurological | Shotgun | Serology | Identification of hepatitis E virus in a lung-transplant patient with meningoencephalitis; examination of patient records indicated it unlikely they were infected prior to transplantation; subsequent testing of donor serum indicated presence of IgG and IgM to HEV but negative for RNA; patient was ultimately treated with antivirals | 2017 | [96] |
| Neurological | Shotgun | Serology; Histopathology | Identification of powassan virus in a patient with severe and progressive encephalitis; patient was discharged to an acute care facility with minimal neurological recovery | 2017 | [106] |
| Neurological | Shotgun | Serology | Identification of west nile virus in a patient with meningoencephalitis; preliminary results were reported to the treating clinicians 9 days post hospital discharge | 2017 | [93] |
| Neurological | Shotgun | RT-PCR; Immunohistochemistry | Identification of cache valley virus in a patient with chronic meningoencephalitis; this diagnosis reinforced a switch from using intravenous immunoglobulin G (IVIg) from Australian donors (where CVV is not found) to IVIg from American donors; the change in treatment did not arrest the patient’s decline and eventual death 42 months after initial presentation | 2017 | [99] |
| Ocular | Shotgun | N/A | Identification of *Brugia malayi* in a patient with extensive itching, swelling and erythema; the patient and her father (experiencing less intense symptoms) were given targeted treatment and three months later the patient's symptoms had resolved | 2016 | [132] |
| Respiratory | Shotgun | Serology | Identification of varicella zoster virus reactivation in a patient with a sore throat and undergoing treatment with Fingolimod; Fingolimod was discontinued and the patient was successfully treated with Acyclovir | 2016 | [73] |
| Respiratory | Shotgun | RT-PCR | Metagenomics was used in a case of Hospital Infection Protocol to identify a link between 3 cases of Human Parainfluenza Virus 3; educational material on the transmission of HPI virus was disseminated to hospital staff; all three patients’ symptoms resolved. | 2017 | [33] |
| Respiratory | Shotgun; Targeted 16S rRNA | Culture | The study demonstrates that in principle, metagenomics accelerates pathogen identification, improving delivery of tailored therapies. | 2017 | [77] |
| Respiratory | Shotgun | Serology | Identification of human rhino virus B91 in a patient with severe pneumonia; the patient improved after targeted treatment and was discharged on day 18 | 2017 | [78] |
| Urinary tract | Targeted 16S rRNA | Urinalysis; Culture | Metagenomics detected a complex bacterial profile with fastidious and anaerobic bacteria, in the urine of a patient with persistent urinary symptoms; antibiotic treatment was unable to resolve the patient's symptoms, though the identification of *Ureaplasma* was thought to be causing the chronic symptoms, this remained to be established | 2014 | [134] |
| **Proof-of-concept** | | | | | |
| Cardiac and bloodstream | Shotgun | Virochip | NA | 2012 | [119] |
| Gastrointestinal | Shotgun | Conventional parasitology; rapid diagnostic testing; Luminex GPP | NA | 2016 | [126] |
| Joint | Shotgun | Culture | NA | 2017 | [137] |
| Joint | Shotgun | Culture | NA | 2017 | [139] |
| Neurological | Shotgun | Isothermal linear nucleic acid amplification (Ribo-SPIA, NuGen) | NA | 2015 | [105] |
| Neurological | Shotgun | Pathology | NA | 2016 | [107] |
| Ocular | Shotgun | PCR | NA | 2016 | [130] |
| Other | Shotgun | PCR; Immunohistochemical analysis; Serology | NA | 2008 | [140] |
| Other | Shotgun | Culture | NA | 2017 | [141] |
| Respiratory | Shotgun | NA | NA | 2011 | [69] |
| Respiratory | Shotgun | Culture; AST | NA | 2016 | [74] |
| Respiratory | Shotgun | Conventional microbiologic assays | NA | 2017 | [91] |
| Respiratory | Shotgun | Culture; PCR | NA | 2017 | [12] |
| Respiratory | Shotgun; Targeted 16S rRNA | Culture | NA | 2017 | [85] |
| Respiratory | Shotgun | Culture; PCR; Serology; Pan-viral group PCR | NA | 2017 | [80] |
| Respiratory | Shotgun | Luminex xTAG viral panel | NA | 2017 | [81] |
| Urinary tract | Targeted 16S rRNA | Culture; PCR | NA | 2008 | [149] |
| Urinary tract | Shotgun | Culture; WGS | NA | 2017 | [135] |
| Urinary tract | Shotgun | Culture; WGS; MALDI-TOF | NA | 2017 | [136] |
| **Retrospective** | | | | | |
| Cardiac and bloodstream | Shotgun | PCR | NA | 2014 | [112] |
| Cardiac and bloodstream | Targeted 16S rRNA | Culture; AST | NA | 2016 | [115] |
| Cardiac and bloodstream | Shotgun | Culture | NA | 2016 | [116] |
| Cardiac and bloodstream | Shotgun | RT-PCR; Serology | NA | 2016 | [118] |
| Cardiac and bloodstream | Targeted 16S rRNA | None mentioned | NA | 2017 | [114] |
| Gastrointestinal | Shotgun | RT-PCR | NA | 2008 | [150] |
| Gastrointestinal | Shotgun | PCR | NA | 2008 | [151] |
| Gastrointestinal | Shotgun | Culture | NA | 2013 | [152] |
| Gastrointestinal | Shotgun; Targeted 16S rRNA | PCR; Real-time PCR; Culture; WGS | NA | 2016 | [125] |
| Neurological | Shotgun | PCR; 16S rRNA PCR | NA | 2016 | [103] |
| Ocular | Shotgun | PCR | NA | 2017 | [131] |
| Ocular | Shotgun | RT-PCR | NA | 2017 | [133] |
| Respiratory | Shotgun | PCR; RT-PCR | NA | 2009 | [68] |
| Respiratory | Shotgun | PCR | NA | 2015 | [71] |
| Respiratory | Shotgun | RT-PCR | NA | 2016 | [72] |
| Respiratory | Shotgun; Targeted 16S rRNA | PCR | NA | 2016 | [75] |
| Respiratory | Shotgun | Culture; Luminex xTAG respiratory panel | NA | 2016 | [76] |
| Respiratory | Shotgun | RT-PCR | NA | 2017 | [90] |
| Respiratory | Shotgun | PCR | NA | 2017 | [79] |
| Respiratory | Shotgun | RT-PCR | NA | 2017 | [82] |
